# The Cap Snatching of Segmented Negative Sense RNA Viruses as a Tool to Map the Transcription Start Sites of Heterologous Co-infecting Viruses

**DOI:** 10.3389/fmicb.2017.02519

**Published:** 2017-12-14

**Authors:** Wenzhong Lin, Ping Qiu, Jing Jin, Shunmin Liu, Saif Ul Islam, Jinguang Yang, Jie Zhang, Richard Kormelink, Zhenguo Du, Zujian Wu

**Affiliations:** ^1^Fujian Province Key Laboratory of Plant Virology, Plant Protection College, Fujian Agriculture and Forestry University, Fuzhou, China; ^2^Tobacco Research Institute, Chinese Academy of Agricultural Sciences, Qingdao, China; ^3^Laboratory of Virology, Wageningen University and Research Centre, Wageningen, Netherlands; ^4^State Key Laboratory of Ecological Pest Control for Fujian and Taiwan Crops, Fuzhou, China

**Keywords:** cap-snatching, sNSV, tenuivirus, TSS, geminivirus, rice stripe virus, co-infection

## Abstract

Identification of the transcription start sites (TSSs) of a virus is of great importance to understand and dissect the mechanism of viral genome transcription but this often requires costly and laborious experiments. Many segmented negative-sense RNA viruses (sNSVs) cleave capped leader sequences from a large variety of mRNAs and use these cleaved leaders as primers for transcription in a conserved process called cap snatching. The recent developments in high-throughput sequencing have made it possible to determine most, if not all, of the capped RNAs snatched by a sNSV. Here, we show that rice stripe tenuivirus (RSV), a plant-infecting sNSV, co-infects *Nicotiana benthamiana* with two different begomoviruses and snatches capped leader sequences from their mRNAs. By determining the 5′ termini of a single RSV mRNA with high-throughput sequencing, the 5′ ends of almost all the mRNAs of the co-infecting begomoviruses could be identified and mapped on their genomes. The findings in this study provide support for the using of the cap snatching of sNSVs as a tool to map viral TSSs.

## Introduction

Messenger RNAs (mRNAs) in a eukaryotic cell normally contain a co-transcriptionally attached 7-methylguanosine (^m7^G) cap at their 5′ termini (Shatkin, 1976). This cap plays important roles in translation by serving as a recognition site for translation initiation factors (Wilkie et al., 2003). All viruses depend on the host translational machinery to produce their proteins (Raoult and Forterre, 2008). Thus, with the exception of some viruses that have developed translation strategies independent from a 5′-cap structure, viruses also need to cap their own mRNAs. Most viruses do so by means of a viral methyl transferase (MT) encoded within their polymerases (Kneller et al., 2006; Decroly et al., 2011). However, segmented negative-sense RNA viruses (sNSVs) of the order *Bunyavirales* and the families *Orthomyxoviridae* and *Arenaviridae* do not contain MT activity within their polymerases and have evolved a unique mechanism called cap snatching to cap their mRNAs (Decroly et al., 2011; Reich et al., 2014). During this process, sNSVs cleave cellular mRNAs at a site 10–15 nt downstream of the cap and use the resultant capped RNA fragment as a primer to initiate transcription of their viral genome. The viral mRNAs synthesized in this way thereby are being discriminated from the viral (anti)genomic templates by the presence of a non-viral host-derived 5′ leader sequence (Decroly et al., 2011). It seems that all capped RNAs within a host cell can serve as cap donors and sNSVs select these donors largely based on their abundance. The recent developments in high-throughput sequencing have made it possible to determine most, if not all, of the capped RNAs snatched by a sNSV (Sikora et al., 2014, 2017; Gu et al., 2015; Koppstein et al., 2015; Liu et al., 2017).

Geminiviruses constitute a family of plant viruses with a circular single-stranded DNA (ssDNA) genome (Hanley-Bowdoin et al., 1999). Begomoviruses, which are transmitted by whiteflies and infect dicotyledonous plants, forms the largest and economically most important genus of the family *Geminiviridae* (Varma and Malathi, 2003). The genome of a begomovirus can be either monopartite or bipartite. The two ssDNA components of a bipartite begomovirus are called DNA-A and DNA-B, respectively, both of which are about 2.7–3 kb in size. DNA-A contains five or six open reading frames (ORFs), one or two on the virion strand (AV1 and AV2) and four on the complementary strand (AC1–AC4). DNA-B contains two ORFs, one each on the virion (BV1) and complementary (BC1) strand, respectively. The ORFs specified by DNA-A overlap with each other extensively, with AC4 embedded totally within AC1. Monopartite begomoviruses have genomes consisting of a single circular ssDNA component that is equivalent to the DNA-A of bipartite begomoviruses. However, many monopartite begomoviruses are associated with DNA satellites known as alphasatellites or betasatellites (Hanley-Bowdoin et al., 1999; Nawaz-ul-Rehman and Fauquet, 2009).

Geminiviruses do not have proteins dedicated to transcription and depend on host RNA polymerase II to produce their mRNAs (Hanley-Bowdoin et al., 1999). These viruses therefore have become attractive models to investigate transcription regulation mechanisms of plants (Hanley-Bowdoin et al., 1999). For some begomo- or geminiviruses, the mRNA 5′ termini have been determined. Initially, this was done by S1 nuclease protection assays followed by primer extension experiments, which are time-consuming and labor-intensive (Townsend et al., 1985; Petty et al., 1988; Hanley-Bowdoin et al., 1989; Sunter and Bisaro, 1989; Sunter et al., 1989; Mullineaux et al., 1993). Recently, the development and commercialization of methods collectively known as rapid amplification of cDNA ends (RACE) have made this easier (Wang and Scott Young, 2003; Shivaprasad et al., 2005; Akbar et al., 2012). However, even with these modern (costly) methods, the mapping of the 5′ ends of all viral mRNAs of a begomovirus is still not a trivial task and involves the optimization of many primer and reaction conditions.

Many sNSVs are able to snatch capped-leader sequences from mRNAs of a co-infecting virus. This observation has led researchers in the past to employ the approach of mixed infections to study the mechanism of cap-snatching (Estabrook et al., 1998; Duijsings et al., 1999; Yao et al., 2012; Liu et al., 2016). In this study, we moved a step forward to show that we could exploit this phenomenon to map the transcription start sites (TSSs) of heterologous co-infecting viruses, with two begomoviruses as examples. Our results provide support for the using of the cap snatching of sNSVs as a tool for molecular studies.

## Results

### RSV co-infects *Nicotiana benthamiana* with different begomoviruses

To test whether it would be possible to exploit the mechanism of cap snatching for the mapping of begomoviral TSSs to their corresponding genome DNA templates, we first verified the occurrence of mixed infections of tenuiviruses and begomoviruses. To this end, we selected the rice stripe tenuivirus (RSV) as the cap-snatching sNSV (Falk and Tsai, 1998), as this virus was earlier shown to snatch capped RNA fragments from a co-infecting cucumber mosaic virus (CMV) in *N. benthamiana* and from a co-infecting rice ragged stunt virus (RRSV) in rice (Yao et al., 2012; Liu et al., 2016). For begomoviruses, two evolutionarily distant species, tomato yellow leaf curl virus (TYLCV) and ramie mosaic virus (RaMV), were selected (Navot et al., 1991; Li et al., 2010).

**Figure 1:**
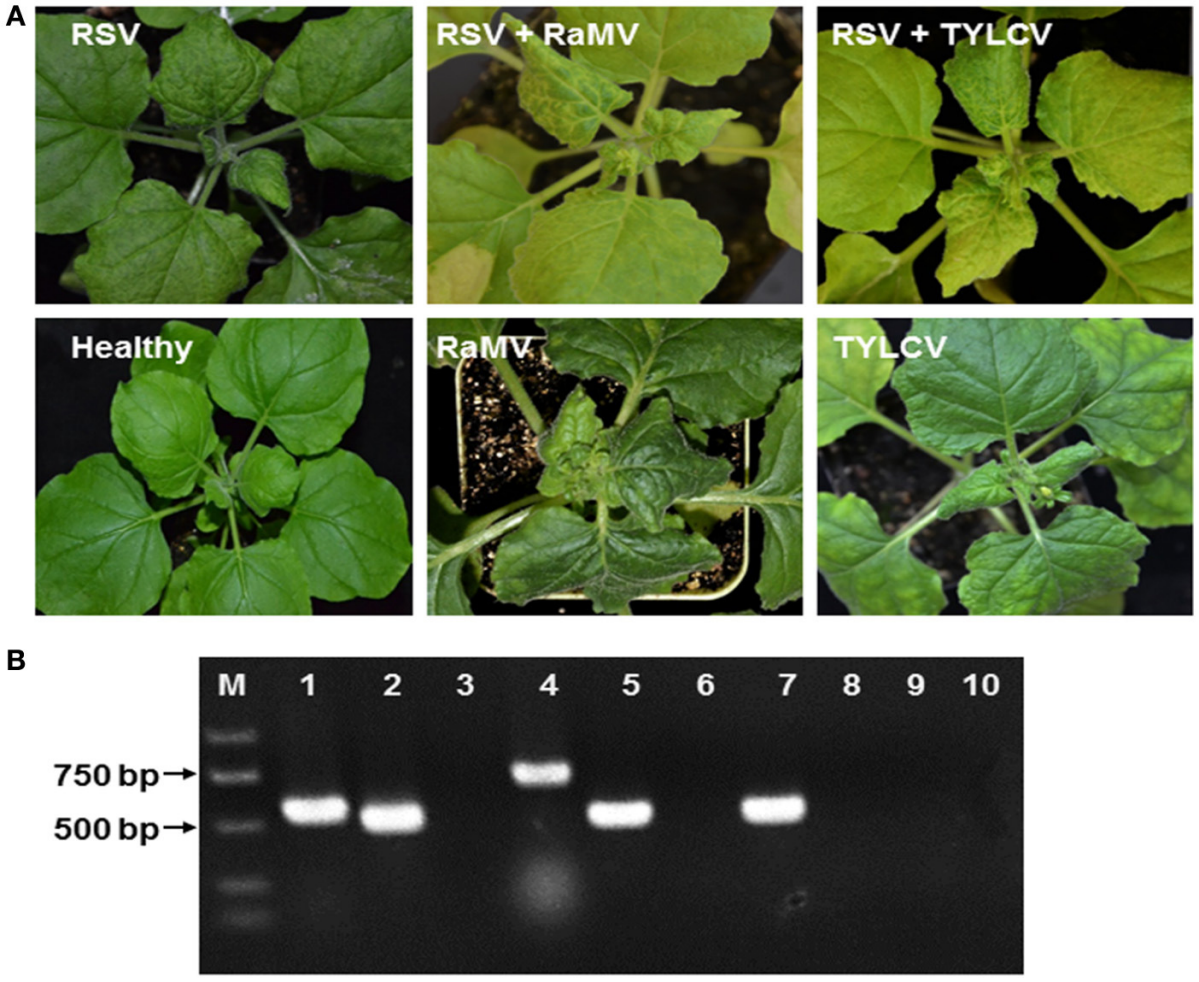
**(A)** Symptoms of *Nicotiana benthamiana* infected by rice stripe tenuivirus (RSV) or co-infected by RSV and tomato yellow leaf curl virus (TYLCV) or RSV and ramie mosaic virus (RaMV), as indicated. *N. benthamiana* infected by either of the two begomoviruses alone were shown for comparison. The *N. benthamiana* were photographed 15 (double infection) or 19 (plants infected by a begomovirus alone) days post inoculation of the begomoviruses. **(B)** Detection of mixed infections by (RT)-PCR. RNA or DNA was extracted from healthy *N. benthamiana* (lanes 3, 6, and 10), *N. benthamiana* infected by RSV alone (lanes 7–9) *N. benthamiana* doubly infected by RSV and RaMV (lanes 1 and 2) or *N. benthamiana* doubly infected by RSV and TYLCV (lanes 4 and 5). RT-PCR was used to detect RSV (lane 2, 5, 7, and 10) and PCR was used to detect RaMV (lanes 1, 3, and 8) and TYLCV (lanes 4, 6, and 9) with virus-specific primers. M, molecular maker.

RSV-infected *N. benthamiana* were obtained by mechanical inoculation and those that became infected were next super-infected with a begomoviral infection by agro-infiltration of infectious clones. In all cases, we observed a mixed infection of RSV and the begomovirus of interest, although the time of appearance of the symptom varied (Figure 1A). Total RNA and DNA were extracted from upper leaves of these plants and the presence of RSV and the begomovirus in all mixed infections was confirmed using RT-PCR/PCR (Figure 1B).

### RSV snatches capped RNA leaders from begomoviral mRNAs

**Figure 2:**
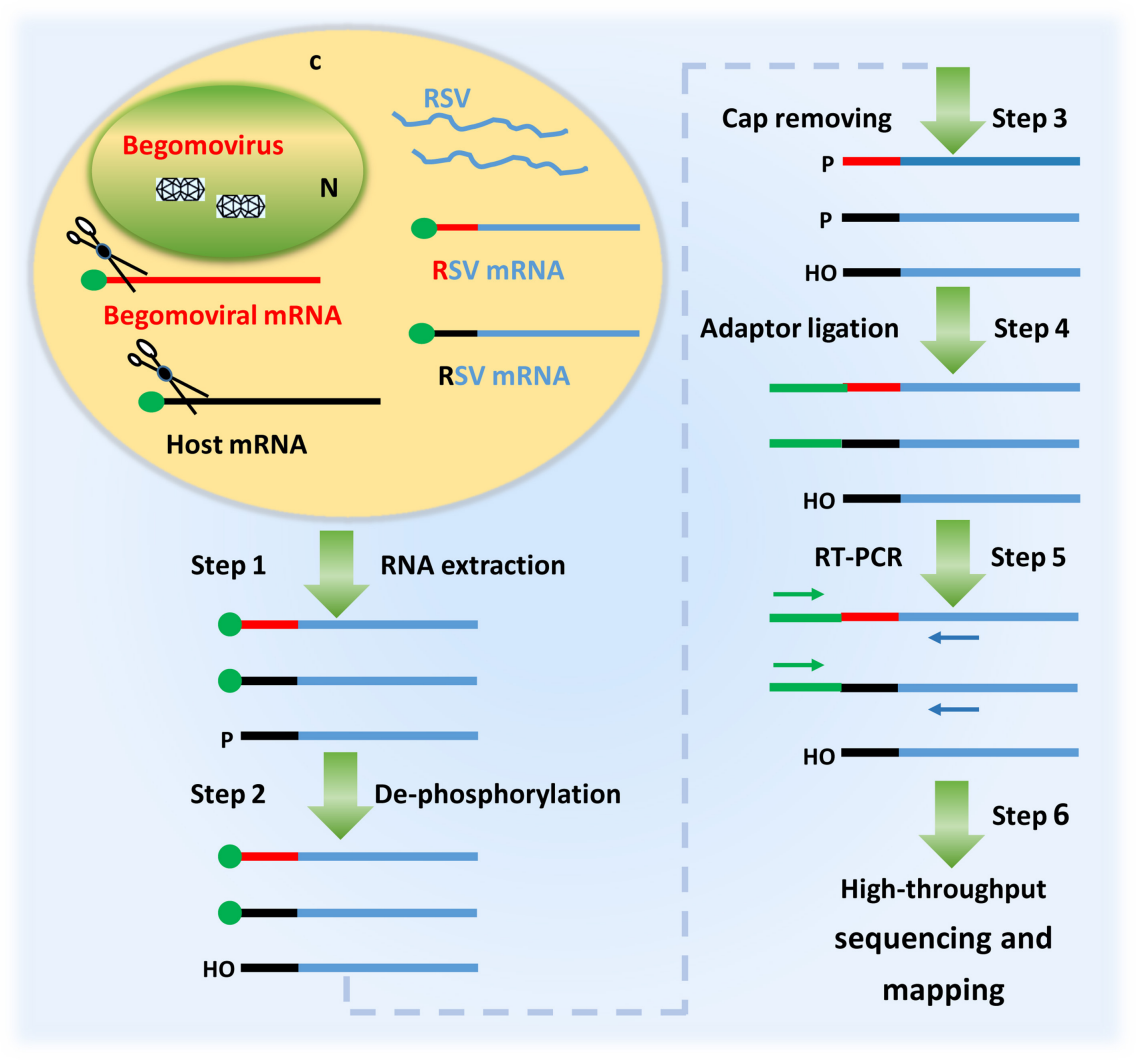
A diagram showing the procedure of identifying the 5′ termini of begomoviral mRNAs using the cap snatching of rice stripe tenuivirus (RSV). In a co-infected host cell, RSV snatches capped RNA leaders from host mRNAs (black) as well as those of the co-infecting begomovirus (red). RSV mRNAs (blue) with heterogeneous 5′ terminal sequences were extracted from the co-infected host plant (step 1); the RNA was treated with alkaline phosphatase to remove the 5′-phosphate groups of some RNA species (step 2) and RppH (NEB) to de-cap mRNAs and leave a monophosphate at their 5′ ends (step 3); a RNA oligo was added to the 5′ monophosphate-bearing mRNAs (step 4) and the oligo-tagged mRNAs were reverse transcribed and PCR amplified (Step 5); The PCR products were used for library construction and high-throughput sequencing. The primer sequences obtained were mapped to the genome of a begomovirus (Step 6).

Having observed that RSV was able to co-infect *N. benthamiana* with TYLCV and RaMV, we next verified whether RSV mRNAs contained 5′ capped leader sequences derived from the mRNAs of the two begomoviruses. Considering that RSV, besides begomoviral mRNAs, uses a large variety of host-cellular capped RNA leaders to prime production of its viral mRNAs (Liu et al., 2017), we aimed to collect RSV viral transcript sequences as much as possible. To this end, a high-throughput sequencing procedure as diagramed in Figure 2 was established and aimed at the 5′ ends of RSV *noncapsid protein (NCP)* transcripts, a mRNA that is being produced in relatively large amounts in RSV infected plant cells (Zhu et al., 1992). Two plants, one co-infected with RSV and RaMV (Sample 1) and the other one co-infected with RSV and TYLCV (Sample 2) were used for the sequencing. Another sample (Sample 3), infected with RSV only, was used as a (negative) control.

For each sample, about 1.2 million sequences were obtained. The sequences were compared with the genome of the co-infecting begomoviruses to extract *NCP* cDNA clones with a 5′ terminal capped leader snatched from the begomovirus. In total, 53 and 30 distinct *NCP* sequences with their 5′ termini matching the genome of RaMV and TYLCV, respectively, were collected. These sequences, along with their read numbers are presented in Table 1 and Supplemental Table 1. Collectively, RaMV- and TYLCV-matching sequences accounted for 2 and 0.5% of the total reads of Sample 1 and Sample 2, respectively. Most of these sequences were found exclusively in either Sample 1 or Sample 2, collected from leaf material containing a mixed-infection, and were absent from Sample 3, collected from singly, RSV-infected leaf material, indicating that these 5′ capped-RNA leader sequences were derived from transcripts of the co-infecting begomovirus (Table 1 and Supplemental Table 1).

**Table 1:**
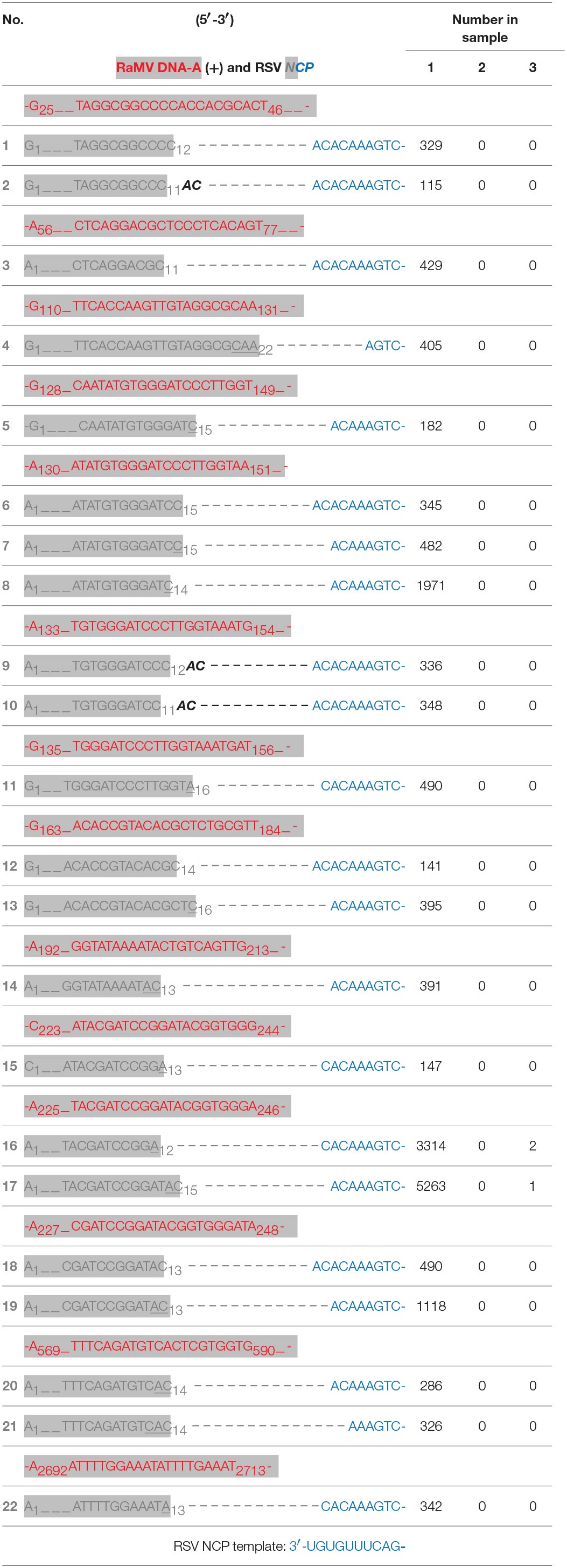
Mapping the cDNA clones of rice stripe tenuivirus (RSV) *NCP* (numbered) to the virion strand of ramie mosaic virus (RaMV) DNA-A (red and shaded).

*RSV NCP mRNA 5′ capped leader sequences matching RaMV genome strand of DNA-A are in grey and shaded; RSV NCP cDNA region matching the template (bottom row) is in blue; RSV NCP cDNA 5′ terminal residues that may be generated by the prime-and-realign mechanism are in black and italicized. The numbering of nucleotides of RaMV is relative to the last A residue of the geminivirus-conserved nanonucleotide sequence TAATATTAC (being numbered nt 1); Dotted lines were introduced to align the sequences or to represent omitting residues*.

According to a current model supported by an increasing number of studies, sNSVs snatch capped-RNA leader sequences from cellular mRNAs in a certain size range to prime viral genome transcription by base-pairing with the 3′ ultimate residues of the viral RNA template. In some cases, the transcription stalls and the elongated capped-RNA leader shifts backwards to realign on the 3′ ultimate residues of the viral RNA template, a process commonly known as prime-and-realign (Garcin et al., 1995; Liu et al., 2016). All the *NCP* transcript sequences in Table 1 and Supplemental Table 1 clearly fit this model. E.g., the first *NCP* sequence can be explained by a cleavage of a putative RaMV mRNA after the residue C_12_. The resulting capped-RNA leader, 5′-G_1_UAGGCGGCCC_11_C_12_, base-pairs by its 3′-ultimate C_12_ residue with G_2_ of the RSV *NCP* template (which has a 3′-U_1_G_2_U_3_G_4_U_5_U_6_UCAG- sequence at its 3′ terminus, see the bottom row of Table 1). After an extension of two residues according to the template, the extended capped-RNA leader, 5′-G_1_UAGGCGGCCC_11_C_12_A_13_C_14_, is shifted back to pair with its A_12_C_13_ residues to U_1_G_2_ of the RSV template. After that, a processive elongation starting from U_3_ gives rise to the *NCP* mRNA. The second sequence can be explained similarly, except that the putative mRNA was cleaved after C_11_ and two cycles of priming and realignment occurred before processive transcription elongation resulting in the addition of a repetitive AC dinucleotide after the snatched capped-RNA leader. Other sequences can be explained in a similar way except that different RaMV mRNAs have been used as donors cap leaders.

**Figure 3:**
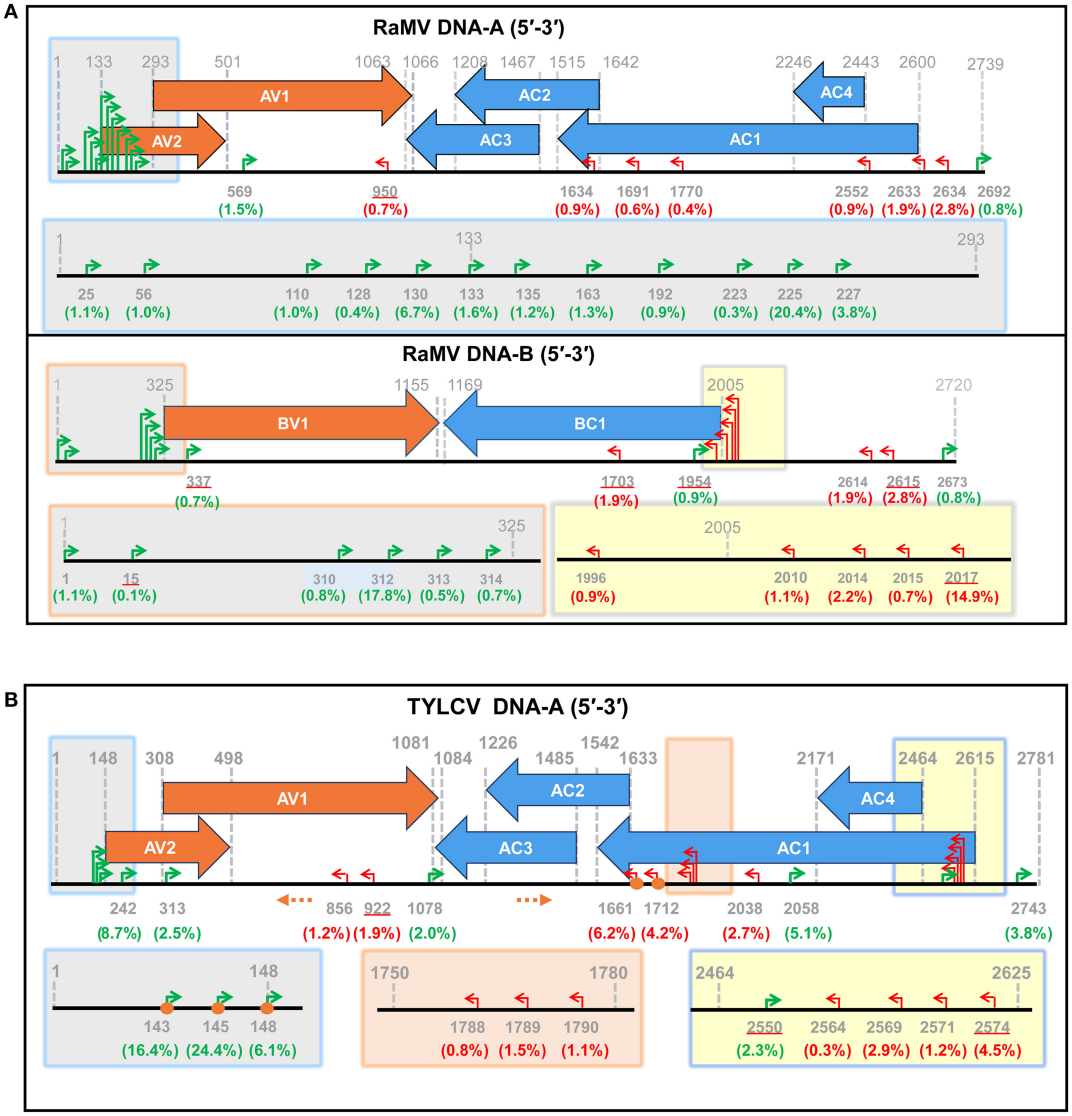
Identification of putative begomoviral transcriptional sites (TSSs) by mapping of the capped RNA leader sequences on the genome of ramie mosaic virus (RaMV) **(A)** and tomato yellow leaf curl virus (TYLCV) **(B)**. The horizontal arrows indicate the open reading frames (ORF) and their directions. The coordinates for TSSs and ORFs are given in green (rightward transcription events), red (leftward transcription events) or gray (ORF boders). The percentages of reads pointing to particular TSSs (number of reads pointing to a particular TSS/ number of reads mapping to a particular virus) were shown in brackets below the nt coodinates. TSSs that are also matched by sequences from Sample 3 are underlined. The shaded boxes on the genome maps are enlarged below. Orange circles indicate TSSs that were verified by traditional RACE. Orange dotted arrows indicate the position and direction of the PCR primers used in RACE. The genome of the two begomoviruses is drawn roughly to scale. The numbering of nucleotides of begomoviral genome fragments is relative to the last A residue of the geminivirus-conserved nanonucleotide sequence TAATATTAC (being numbered nt 1); For complementary DNA fragments, the coordinates of the complementary virion-sense nucleotides were given.

Following this result, we next set out to map the 5′ ends of these putative begomoviral mRNAs to the genome of RaMV or TVLCV. As shown and summarized in Figure 3, mapping of the snatched RaMV and TYLCV 5′ capped-RNA leaders on their corresponding DNA genomes revealed transcriptional start sites (TSSs) of geminiviral genes that were largely in agreement with those reported from previous studies on related viruses. Notably, the heterogeneity of the TSSs identified here was considerably higher than that previously observed with primer extension experiments (Townsend et al., 1985; Petty et al., 1988; Hanley-Bowdoin et al., 1989; Sunter and Bisaro, 1989; Sunter et al., 1989; Mullineaux et al., 1993), and more or less comparable to (or slightly higher than) that identified recently with 5′ RACE experiments (Shivaprasad et al., 2005; Akbar et al., 2012). In addition, a putative TSS at nt 1,634 of RaMV was found. This TSS suggested the presence of a monocistronic AC3 mRNA (Figure 3A and Supplemental Table 1). This is somewhat surprising since AC3 is considered to be expressed from a polycistronic mRNA according to the results obtained previously for all begomoviruses except mungbean yellow mosaic virus (MYMV, Shivaprasad et al., 2005).

### Verification of the 5′ termini of begomoviral mRNAs

**Figure 4:**
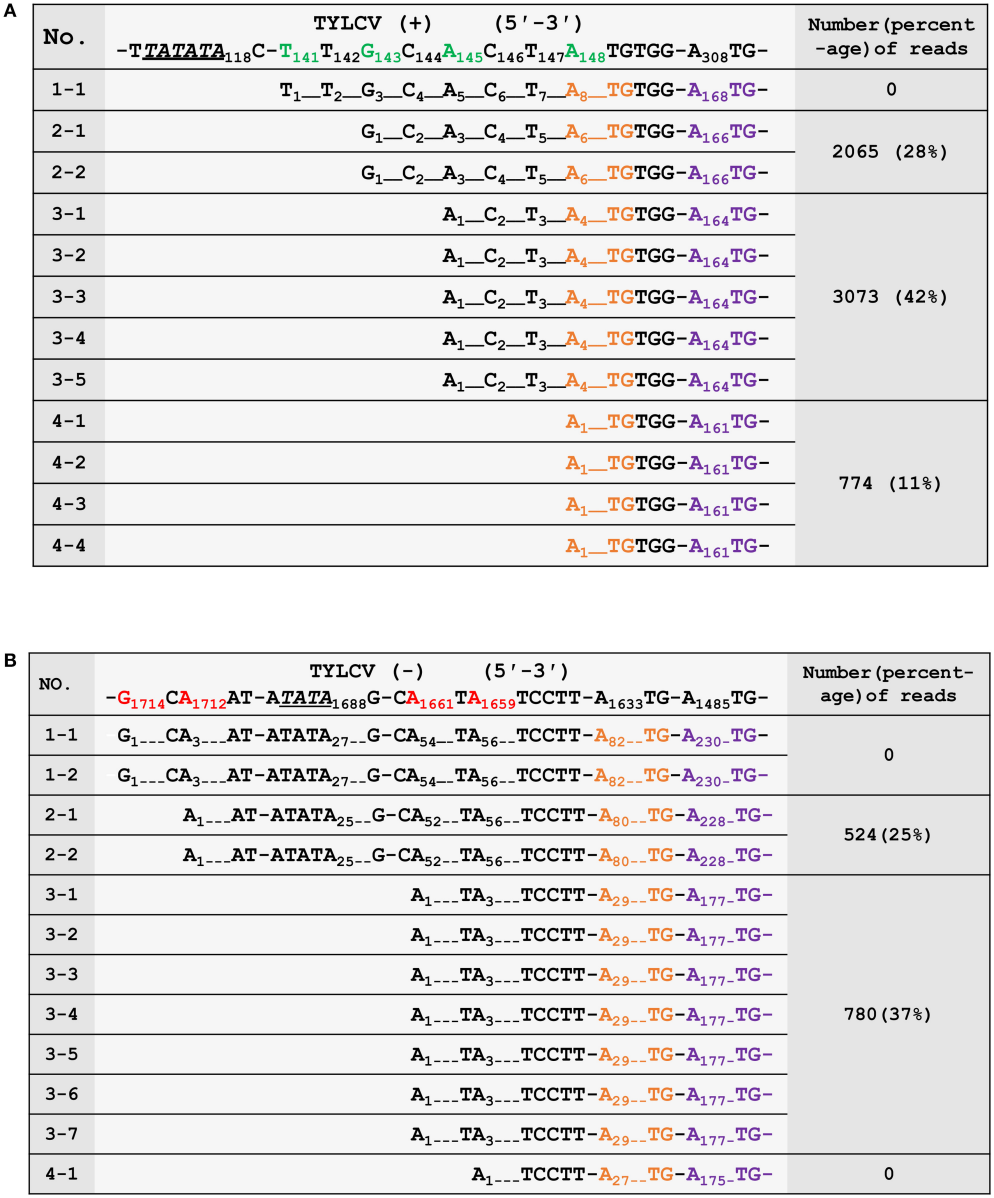
Verification of the 5′ termini of mRNAs from the virion- **(A)** and complementary-strand **(B)** of tomato yellow leaf curl virus (TYLCV). The first row shows the genomic sequence of TYLCV. The transcriptional start sites (TSSs) are in green (virion strand) or red (complementary strand). Putative TATA boxes about 30 nt upstream of the TSSs are underlined and italicized. The nucleotides corresponding to the ATG start codons of AV2/AC2 and AV1/AC3 are in orange and purple, respectively. The third column shows the number and percentages of high-throughput sequencing reads in Sample 2 (number of reads pointing to a particular TSS/ number of reads pointing to TSSs upstream of the RACE primer) that have identified the same TSSs. The numbering of nucleotides of begomoviral genome fragments is relative to the last A residue of the geminivirus-conserved nanonucleotide sequence TAATATTAC (being numbered nt 1); For complementary DNA fragments, the coordinates of the complementary virion-sense nucleotides were given. Dotted lines were introduced to align the sequences or to represent omitting residues.

To confirm the 5′ termini of the begomoviral mRNAs obtained from the cap-snatching based cloning strategy, an RNA ligase mediated 5′ RACE method was applied to collect RSV transcript sequences and analyze their heterogeneous 5′ leader sequences (Liu and Gorovsky, 1993). The procedure of this method is similar to that shown in Figure 2. In brief, an adaptor was added to the 5′ termini of capped RNAs. The 5′ oligo-tagged RNAs were reverse transcribed with gene-specific primers and subsequently PCR amplified with a primer specific to the adaptor (forward primer) and two different reverse primers (the positions of which are indicated in Figure 3B) specific to virion- or complementary-sense transcripts of TYLCV, respectively. Both PCR reactions yielded multiple fragments of different sizes that were all purified and cloned for sequence analyses. As shown in Figures 4A,B, 12 sequences were obtained for each PCR reaction, and mapping them on the viral DNA genome of TYLCV pointed toward four TSSs for virion-sense transcripts, i.e., at nt 141, 143, 145, and 148 of the viral genome (Figure 4A), and also four for the complementary-sense transcripts, i.e., at nt 1,659, 1,661, 1,712, and 1,714, respectively (Figure 4B). Of the four virion-sense TSSs, three (at nt 143, 145, and 148, respectively) were found in the cap-snatching based cloning strategy (Figure 3B and Supplemental Table 1). For the RACE, 11 of the 12 (92%) cDNA clones pointed toward these three TSSs. For the cap-snatching based cloning strategy, 5,912 of the 7,326 (81%) expected reads (reads matching TYLCV genome regions upstream of the primer used in the RACE) pointed toward these three TSSs. Of the four complementary-sense TSSs, two (at nt 1,712 and 1,661, respectively) were identical to those identified by the cap-snatching based cloning strategy (Figure 3B and Supplemental Table 1). For the RACE, 9 of the 12 (75%) cDNA clones pointed toward these three TSSs. For the cap-snatching based cloning strategy, 1,304 of the 2,082 (63%) expected reads pointed toward these three TSSs. While one virion- and two complementary-sense TSSs were identified exclusively by RACE, two virion- and four complementary-sense TSSs were identified only by the cap-snatching based cloning strategy. These results altogether suggested that the cap-snatching based cloning strategy enabled the mapping and identification of not only most commonly used TSSs but also some rare ones.

## Discussion

In the past two decades, several studies have described the use of mixed viral infections to study the mechanism of cap snatching for several plant-infecting sNSVs (Estabrook et al., 1998; Duijsings et al., 1999; Yao et al., 2012; Liu et al., 2016). Here we showed the feasibility of using this phenomenon to map the mRNA TSSs of a heterologous co-infecting virus. Besides tenuiviruses, two other groups of plant viruses, i.e., tospoviruses and emaraviruses, use the cap-snatching mechanism (Jin and Elliott, 1993; Walia and Falk, 2012). The three groups of plant viruses as a whole cover a very large host range (Kormelink et al., 2011) and thereby provide the possibility to apply the cap-snatching-based 5′-end transcript mapping approach to almost any virus that is able to co-infect with one of these viruses in any plant host. Although here showed by using a plant virus, we believe this approach to be generic and applicable to animal-infecting viruses as well due to the highly conserved nature of the cap-snatching mechanism and the ability of sNSVs in the order *Bunyavirales* and the families *Orthomyxoviridae* and *Arenaviridae* to infect a wide range of animals (Decroly et al., 2011). In this sense, it is noteworthy that DNA viruses, some with very large and complex genomes, are much more frequently found in animals compared to plants, which makes our approach for these systems more attractive (King et al., 2012).

So far, the 5′ ends of viral/host mRNAs have been mapped by various molecular techniques, but the most recent ones involve 5′ RACE. During conventional 5′ RACE experiments, primers have to be designed and experimental conditions optimized for each mRNA. For viruses with a complex ORF arrangement such as a begomovirus, *a priori* knowledge on the approximate locations of the TSSs is needed to design proper primers. In addition, one has to sequence a big number of clones to gain a full appreciation of the heterogeneity of the TSSs of a virus. With the approach established here, all what needs to be done is to sequence the 5′ ends of a single sNSV mRNA because the sNSV has gathered the 5′ ends of all the mRNAs of interest. In contrast to traditional RACE experiments in which the 5′ ends of different begomoviral mRNAs are obtained in different reactions, the procedure established here allows the identification of the 5′ ends of different mRNAs of a begomovirus in the same sequencing reaction. This makes it possible to have a global evaluation on the abundance of each transcript of a begomovirus, although caution should be taken because sNSVs may have a preference for some mRNAs (Geerts-Dimitriadou et al., 2011a,b). Furthermore, since we do not have to determine the 5′ termini of assumed mRNAs with specially designed primers, our new (more unbiased) approach has the potential to identify novel and unexpected mRNAs of a begomovirus. In this respect, the results from our study did reveal the presence of occasional leftward transcripts on virion-sense DNA and rightward transcripts on complementary-sense DNA for both RaMV and TYLCV, although further experiments are needed to verify these findings (Figure 3). Last but not least, our newly established approach perfectly combines with high-throughput sequencing, a technology that has faced great price drops in recent years.

One major drawback with this new approach is that the 5′-capped leader sequences snatched by a sNSV are generally not more than 15 nt in size (Table 1 and Supplemental Table 1; Sikora et al., 2014, 2017; Gu et al., 2015; Koppstein et al., 2015). Determining the source of these small-sized fragments may sometimes be ambiguous, i.e., some begomovirus-matching 5′-capped leader sequences could also be derived from unknown host mRNAs (Supplemental Table 1). However, such a problem can be solved by performing in parallel control experiments, as it is shown here. In addition, when a common host plant is used, a shared database (containing host derived 5′ leader sequences) can be built for filtering out such sequences from newly obtained datasets. Another reason for caution concerns the application of a PCR amplification during step 5 of the entire procedure (Figure 2). The number of PCR cycles influences the complexity of the data obtained and might generate some bias, because an over amplification may decrease the chance of obtaining leader sequences that are present in low amounts. For example, we used 34 cycles of PCR in Sample 1 and 2 but 30 cycles of PCR in Sample 3. As a result, the number of unique sequences we got for Sample 3 was more than six times greater than those for Sample 1 and 2, although the numbers of total reads for the three samples were comparable (data not shown). Presumably, because of a low sequencing depth, we did not find the TSS responsible for a transcript of TYLCV AC1 (Figure 3B).

## Materials and methods

### Virus source and inoculation

A Jiangsu isolate of RSV (LS-JSJJ03, EF198702, EF198682, EF198733, EF198702) was used in this study. This virus was maintained in rice by transmission with viruliferous *Laodelphax striatellus* Fallen. The inoculation of RSV to *N. benthamiana* was done as described previously (Zheng et al., 2015). TYLCV and RaMV were isolated from Fujian and maintained with their infectious clones. The RSV infected *N. benthamiana* was super infected with a begomoviral infection by agro-infiltration of infectious clones (10 days after the inoculation of RSV). To do this, *Agrobacterium tumefaciens* strain EHA105 carrying the plasmid of interest were grown to an optical density at 600 nm (OD_600_) of 0.8 at 28°C on LB liquid medium supplemented with rifampicin (50 μg/mL) and kanamycin (50 μg/mL). The cultures were centrifuged at 12,000 g for 1 min followed by resuspension with an induction medium [10 mM 2-(N-morpholino) ethanesulfonic acid (MES), pH 5.6, 10 mM MgCl_2_ and 150 μM acetosyringone]. After virus inoculation, *N. benthamiana* plants were grown under standard greenhouse conditions (16-h day length; 22 to 24°C)

### High throughput sequencing of the 5′ ends of RSV *NCP*

The high throughput sequencing was done with a procedure diagramed in Figure 2. Briefly, total RNA was extracted from *N. benthamiana* 15 days after inoculation of begomoviruses using Triziol (Invitrogen). After being dephosphorylated using alkaline phosphatase (NEB), the total RNA was treated with RppH (NEB), which removes the cap structure of mRNAs and leaves a monophosphate at their 5′ ends. T4 RNA Ligase 1 (NEB) was used to ligate a RNA oligo (TCTACrArGrUrCrCrGrArCrGrArUrC) to the 5′-monophosphate-bearing mRNAs. SuperScript® III First-Strand Synthesis System (Invotrogen) was used to reverse transcribe the oligo-tagged RNAs with a primer named NCPR1, which is specific to *NCP* of RSV. Two primers, one designed according to the RNA oligo (GTTCTACAGTCCGACGATC) and another specific to *NCP* but 5′ to NCPR1, were used to amplify the cDNA. After 30 (Sample 3) or 34 (Sample 1 and 2) cycles of amplification, the PCR products were resolved by agarose gel electrophoresis. PCR bands about 200 bp in size were recovered and sent to Biomarker Technologies (Beijing, China) for high throughput sequencing with a HiSeq 2500 platform.

### Verification of the 5′ termini of begomoviral mRNAs

A RNA ligase mediated RACE (RLM-RACE) was used to verify the 5′ termini of begomoviral mRNAs (Liu and Gorovsky, 1993). Total RNA extraction, dephosphorylation, cap-removing and RNA adaptor ligation were done as described above. Primers TYTAV2 and TYTAC2 were used to reverse transcribe putative virion- and complementary-sense transcripts of TYLCV, respectively (Figure 3B). Primers TYTAV1 and TYTAC1, which are inner to TYTAV1 and TYTAC2, respectively, on corresponding transcripts, were used to PCR amplify the cDNA obtained above, together with a forward primer designed according to the RNA adaptor. The PCR products were resolved by agarose gel electrophoresis and discrete bands were recovered, cloned and sequenced.

## Author contributions

WL and PQ conducted the research. JJ and SU helped in virus inoculation. SL, JZ, and JY helped in sequence library construction. RK helped in data analysis, ZD and ZW conceived and supervised the research.

### Conflict of interest statement

The authors declare that the research was conducted in the absence of any commercial or financial relationships that could be construed as a potential conflict of interest.
