# Supplementary material for: The Cap Snatching of Segmented Negative Sense RNA Viruses as a Tool to Map the Transcription Start Sites of Heterologous Co-infecting Viruses
Source: Front Microbiol. 2017 Dec 14;8:2519. doi: 10.3389/fmicb.2017.02519 (PMC5735111; doi:10.3389/fmicb.2017.02519)
Supplement: Supplementary file 1 [file Table1.DOCX]

**Supplemental Table 1** Mapping of the 5′ capped leader sequences of Rice stripe tenuivirus (RSV) *NCP* mRNAs to the genome of Ramie mosaic virus (RaMV) DNA-A and Tomato yellow leaf curl virus (TYLCV).

|  | **(5′-3′)**  **RaMV DNA-A (-) and RSV *NCP*** |  |  |  |
| --- | --- | --- | --- | --- |
|  | **-A_950-_TTTCCCAGCTTCCTGGTGGTTA_939-_-** |  |  |  |
| **15** | **-A_1---_TTTCCCAGCTTC_13_-----------ACACAAAGTC-** | **278** | **0** | **5** |
|  | **-C_1634_TCTCAAAAGGTGGGCATTACAA_1623_-** |  |  |  |
| **16** | **-C_1---_TCTCAAAAGGTGGGCA_17_----------CAAAGTC-** | **373** | **0** | **0** |
|  | **-A_1691_ATCCAGGCCCAACTTCGTCCTA_1670_-** |  |  |  |
| **17** | **-A_1---_ATCCAGGCCC*AC*_13_-----------ACACAAAGTC-** | **264** | **0** | **0** |
|  | **-C_1770_CAAAGGGACTGGCAAAGCAACA_1749_-** |  |  |  |
| **18** | **-C_1---_CAAAGGGACTGGCA_15_------------CAAAGTC-** | **176** | **0** | **0** |
|  | **-A_2552_CGTATCCTCAGTGCCCTCTCTC_2531_-** |  |  |  |
| **19** | **-A_1---_CGTATCCTCAGTGC_15_-----------ACAAAGTC-** | **368** | **0** | **0** |
|  | **-A_2633_TAGATGGTTGATCCCCGATTGA_2612_-** |  |  |  |
| **20** | **-A_1---_TAGATGGTTGA_12_*C*-----------ACACAAAGTC-** | **304** | **0** | **0** |
| **21** | **-A_1---_TAGATGGTTGA_12_-------------CACAAAGTC-** | **502** | **0** | **0** |
|  | **-G_2634_ATAGATGGTTGATCCCCGATTG_2613_-** |  |  |  |
| **22** | **-G_1---_ATAGATGGTTGA*C*_13_----------ACACAAAGTC-** | **309** | **0** | **0** |
| **23** | **-G_1---_ATAGATGGTTGA_12_------------CACAAAGTC-** | **862** | **0** | **0** |
|  | **(5′-3′)**  **RaMV DNA-B (+) and RSV *NCP*** |  |  |  |
|  | **-A_1---_CCGGATGGCCGCGATTTTTTT_22--_-** |  |  |  |
| **24** | **-A_1---_CCGGATGGC_10_AC**------------**ACACAAAGTC-** | **446** | **0** | **0** |
|  | **-A_15—-_TTTTTTTTTATAGTGGTCCCA_36--_-** |  |  |  |
| **25** | **-A_1---_TTTTTTTTTA_11_AC**-----------**ACACAAAGTC-** | **49** | **0** | **0** |
|  | **-G_310-_CATTGTGAAGTAAAATGAGAA_331-_-** |  |  |  |
| **26** | **-G_1---_CATTGTGAAGTAAAA_16_**---------**CACAAAGTC-** | **317** | **0** | **0** |
|  | **-A_312-_TTGTGAAGTAAAATGAGAATT_333-_-** |  |  |  |
| **27** | **-A_1---_TTGTGAAGTA_11_*C***------------**ACACAAAGTC-** | **273** | **0** | **0** |
| **28** | **-A_1---_TTGTGAAGTAA_12_*C***-------------**ACAAAGTC-** | **345** | **0** | **0** |
| **6** | **-A_1---_TTGTGAAGTA_11_*CAC***----------**ACACAAAGTC-** | **352** | **0** | **0** |
| **29** | **-A_1---_TTGTGAAGTAAAA_14_*C***---------**ACACAAAGTC-** | **474** | **0** | **0** |
| **30** | **-A_1---_TTGTGAAGTAAA_13_**------------**CACAAAGTC-** | **1264** | **0** | **0** |
| **31** | **-A_1---_TTGTGAAGTAAA_13_**-----------**ACACAAAGTC-** | **1782** | **0** | **0** |
| **32** | **-A_1---_TTGTGAAGTA_11_**--------------**CACAAAGTC-** | **2991** | **0** | **0** |
|  | **-T_313-_TGTGAAGTAAAATGAGAATTC_334-_-** |  |  |  |
| **33** | **-T_1---_TGTGAAGTA_10_**--------------**ACACAAAGTC-** | **191** | **0** | **0** |
|  | **-T_314-_GTGAAGTAAAATGAGAATTCC_335-_-** |  |  |  |
| **34** | **-T_1---_GTGAAGTAAA_11_*CACAA***--------**ACACAAAGTC-** | **306** | **0** | **0** |
|  | **-A_337-_TTAGATCTCCTGGTGGGTTTA_358-_-** |  |  |  |
| **35** | **-A_1---_TTAGATCTC_10_*ACAC***----------**ACACAAAGTC-** | **86** | **0** | **0** |
| **36** | **-A_1---_TTAGATCTC_10_*AC***------------**ACACAAAGTC-** | **220** | **0** | **2** |
|  | **-A_1954_CTCTGTCCTGTCTGATGTTGC_1975_-** |  |  |  |
| **37** | **-A_1---_CTCTGTCCTGTC_13_**-------------**ACAAAGTC-** | **171** | **0** | **6** |
| **38** | **-A_1---_CTCTGTCCTGTC_13_**-----------**ACACAAAGTC-** | **205** | **0** | **3** |
|  | **-A_2673_ATTTTGGAAATATTTTGAAAT_2694_-** |  |  |  |
| **39** | **-A_1---_ATTTTGGAAATA_13_**------------**CACAAAGTC-** | **342** | **0** | **0** |
|  | **(5′-3′)**  **RaMV DNA-B (-) and RSV *NCP*** |  |  |  |
|  | **-A_1703_TCATCATACTTCTCACCAAGA_1682_-** |  |  |  |
| **40** | -**A_1---_TCATCATAC_10_*ACAC***----------**ACACAAAGTC-** | **817** | **0** | **2** |
|  | **-A_1996_ACTCGAGTGCATTGGCATATG_1975_-** |  |  |  |
| **41** | -**A_1---_ACTCGAGTGCA_12_**-------------**CACAAAGTC-** | **387** | **0** | **0** |
|  | **-A_2010_CAAGATGGCTGATAACTCGAG_1989_-** |  |  |  |
| **42** | -**A_1---_CAAGATGGCTGATA_15_**----------**CACAAAGTC-** | **481** | **0** | **0** |
|  | **-A_2014_ATCACAAGATGGCTGATAACT_1993_-** |  |  |  |
| **43** | **-A_1---_ATCACAAGATGGC_14_**------------**ACAAAGTC-** | **914** | **0** | **0** |
|  | **-C_2015_AATCACAAGATGGCTGATAAC_1994_-** |  |  |  |
| **44** | **-C_1---_AATCACAAGA_11_**--------------**CACAAAGTC-** | **281** | **0** | **0** |
|  | **-A_2017_TCAATCACAAGATGGCTGATA_1996_-** |  |  |  |
| **45** | **-A_1---_TCAATCACAAGATGGC_17_**---------**ACAAAGTC-** | **308** | **0** | **0** |
| **46** | **-A_1---_TCAATCACAA_11_**--------------**CACAAAGTC-** | **399** | **0** | **185** |
| **47** | **-A_1---_TCAATCACAA_11_*C***------------**ACACAAAGTC-** | **602** | **0** | **356** |
| **48** | **-A_1---_TCAATCACAAGA_13_*C***----------**ACACAAAGTC-** | **1846** | **0** | **0** |
| **49** | **-A_1---_TCAATCACAAGA_13_**------------**CACAAAGTC-** | **3101** | **0** | **0** |
|  | **-A_2614_TAGATGGTTGATCCCCGATTG_2593_-** |  |  |  |
| **50** | **-A_1---_TAGATGGTTGA_12_*C***-----------**ACACAAAGTC-** | **304** | **0** | **0** |
| **51** | **-A_1---_TAGATGGTTGA_12_**-------------**CACAAAGTC-** | **502** | **0** | **0** |
|  | **-G_2615_ATAGATGGTTGATCCCCGATT_2594_-** |  |  |  |
| **52** | **-G_1---_ATAGATGGTTGA_13_*C***----------**ACACAAAGTC-** | **309** | **0** | **0** |
| **53** | **-G_1---_ATAGATGGTTGA_13_**------------**CACAAAGTC-** | **862** | **0** | **0** |
|  |  |  |  |  |
|  | **(5′-3′)**  **TYLCV (+) and RSV *NCP*** |  |  |  |
|  | **-G_143-_CACTATGTGGGATCCACTTCT_164-_-** |  |  |  |
| **1** | **-G_1---_CACTATGTGGGA_13_**------------**CACAAAGTC-** | **0** | **1484** | **0** |
| **2** | -**G_1---_CACTATGTGGGATCCAC_18_**----------**AAAGTC-** | **0** | **581** | **0** |
|  | **-A_145-_CTATGTGGGATCCACTTCTAA_166-_-** |  |  |  |
| **3** | **-A_1---_CTATGTGGGA_11_**--------------**CACAAAGTC-** | **0** | **2075** | **0** |
| **4** | -**A_1---_CTATGTGGGATCCAC_16_**------------**AAAGTC-** | **0** | **577** | **0** |
| **5** | -**A_1---_CTATGTGGGATC_13_**-------------**ACAAAGTC-** | **0** | **421** | **0** |
|  | **-A_148-_TGTGGGATCCACTTCTAAATG_169-_-** |  |  |  |
| **6** | -**A_1---_TGTGGGATCCAC_13_**-------------**ACAAAGTC-** | **0** | **774** | **0** |
|  | **-A_242-_CGAGCCCAATACATTGGGCCA_263-_-** |  |  |  |
| **7** | -**A_1---_CGAGCCCAATACA_14_**-------------**CAAAGTC-** | **0** | **389** | **0** |
| **8** | -**A_1---_CGAGCCCAATACA_14_*C***---------**ACACAAAGTC-** | **0** | **360** | **0** |
| **9** | -**A_1---_CGAGCCCAATAC_13_**-----------**ACACAAAGTC-** | **0** | **348** | **0** |
|  | **-G_313-_AAGCGACCAGGCGATATAATC_334-_-** |  |  |  |
| **10** | -**G_1---_AAGCGACCAGGC_13_**-------------**ACAAAGTC-** | **0** | **317** | **0** |
|  | **-A_1078_AATTAATAAAATTTATATTTT_1101_-** |  |  |  |
| **11** | -**A_1---_AATTAATAAA_11_*CAC***----------**ACACAAAGTC-** | **0** | **247** | **0** |
|  | **-A_2058_GAAGAAGAAAGAAATGGAGA_2081_-** |  |  |  |
| **12** | -**A_1---_GAAGAAGAAA_11_**-------------**ACACAAAGTC-** | **0** | **340** | **0** |
| **13** | -**A_1---_GAAGAAGAAA_11_**--------------**CACAAAGTC-** | **0** | **299** | **0** |
|  | **-A_2550_GAGAGAGAACAATTGGGATAT_2571_-** |  |  |  |
| **14** | -**A_1---_GAGAGAGAA_10_**--------------**ACACAAAGTC-** | **0** | **294** | **26** |
|  | **-A_2743_AAAAATCAAATCATTAAAGCG_2764_-** |  |  |  |
| **15** | -**A_1---_AAAAATCAAA_11_*CAC***----------**ACACAAAGTC-** | **0** | **484** | **0** |
|  | **(5′-3′)**  **TYLCV (-) and RSV *NCP*** |  |  |  |
|  | **-T_856-_TTCCTCATCACTTGAAACCTA_835-_-** |  |  |  |
| **16** | -**T_1---_TTCCTCATCAC_12_**--------------**ACAAAGTC-** | **0** | **156** | **0** |
|  | **-A_922-_AAAAATCTCTTAACTAATGCC_901_-** |  |  |  |
| **17** | -**A_1---_AAAAATCTC_10_**--------------**ACACAAAGTC-** | **0** | **243** | **20** |
|  | **-A_1661_TATCCTTGAAAAATTGGGCTC_1650_-** |  |  |  |
| **18** | -**A_1---_TATCCTTGAA_11_**--------------**CACAAAGTC-** | **0** | **465** | **0** |
| **19** | -**A_1---_TATCCTTGAAA_12_**-------------**CACAAAGTC-** | **0** | **315** | **0** |
|  | **-A_1712_ATCCAGGACCTACCTCCTCAT_1691_-** |  |  |  |
| **20** | **-A_1---_ATCCAGGACC_11_**-------------**ACACAAAGTC-** | **0** | **354** | **0** |
| **21** | **-A_1---_ATCCAGGACCTAC_14_**------------**ACAAAGTC-** | **0** | **170** | **0** |
|  | **-G_1788_AGGGACTGGCAAAGCAACACA_1767_-** |  |  |  |
| **22** | -**G_1---_AGGGACTGGCAAAGCA_17_**----------**CAAAGTC-** | **0** | **101** | **0** |
|  | **-A_1789_GAGGGACTGGCAAAGCAACAC_1768_-** |  |  |  |
| **23** | -**A_1---_GAGGGACTGGCA_13_**--------------**CAAAGTC-** | **0** | **195** | **0** |
|  | **-C_1790_AGAGGGACTGGCAAAGCAACA_1769_-** |  |  |  |
| **24** | -**C_1---_AGAGGGACTGGCAA_15_**----------**CACAAAGTC-** | **0** | **136** | **0** |
|  | **-A_2038_ACTTGAAGAGTGGGTCGCCGA_2517_-** |  |  |  |
| **25** | -**A_1---_ACTTGAAGA_10_*CAC***-----------**ACACAAAGTC-** | **0** | **346** | **0** |
|  | **-A_2564_ATTGTTCTCTCTCTAAAGAGG_2543_-** |  |  |  |
| **26** | -**A_1---_ATTGTTCTCTC_12_**------------**ACACAAAGTC-** | **0** | **42** | **0** |
|  | **-A_2569_TCCCAATTGTTCTCTCTCTAA_2548_-** |  |  |  |
| **27** | -**A_1---_TCCCAATTGTTC_13_**-------------**ACAAAGTC-** | **0** | **368** | **0** |
|  | **-A_2571_TATCCCAATTGTTCTCTCTCT_2550_-** |  |  |  |
| **28** | -**A_1---_TATCCCAATTGTTCTC_17_**---------**ACAAAGTC-** | **0** | **153** | **0** |
|  | **-A_2574_ACATATCCCAATTGTTCTCTC_2553_-** |  |  |  |
| **29** | -**A_1---_ACATATCCCAA_12_*C***-----------**ACACAAAGTC-** | **0** | **408** | **2** |
| **30** | -**A_1---_ACATATCCCAA_12_**------------**ACACAAAGTC-** | **0** | **157** | **1** |
|  | R **RSV NCP template:** **3′-UGUGUUUCAG-** |  |  |  |

For RaMV DNA-A, only the antigenome strand is shown here. RSV NCP mRNA 5′ capped leader sequences matching begomoviral genome fragments are in grey and shaded; RSV NCP cDNA region matching the template (bottom row) is in blue; RSV NCP cDNA 5′ terminal residues that may be generated by the prime-and-realign mechanism are in black and italicized. The numbering of nucleotides of begomoviral genome fragments is relative to the last A residue of the geminivirus-conserved nanonucleotide sequence TAATATTAC (being numbered nt 1); For complementary DNA fragments, the coordinates of the complementary virion-sense nucleotides were given. Dotted lines were introduced to align the sequences or to represent omitting residues.
